# Potassium Titanate Supported Atomically Dispersed Palladium for Catalytic Oxidation

**DOI:** 10.1002/advs.202204674

**Published:** 2022-10-26

**Authors:** Li Zhou, Shuren He, Xiaohong Xu, Guangwu Li, Chuancheng Jia

**Affiliations:** ^1^ Center of Single‐Molecule Sciences Institute of Modern Optics Tianjin Key Laboratory of Micro‐scale Optical Information Science and Technology Frontiers Science Center for New Organic Matter College of Electronic Information and Optical Engineering Nankai University 38 Tongyan Road, Jinnan District Tianjin 300350 P. R. China; ^2^ School of Chemistry and Chemical Engineering Shandong University 27 Shanda Nan Road, Licheng District Jinan Shandong 250100 P. R. China

**Keywords:** catalytic oxidation, metal–support interaction, palladium, potassium titanate, single‐atom catalyst

## Abstract

Single‐atom catalysts based on noble metals provide efficient atomic utilization along with enhanced reactivity. Herein, a convenient strategy to construct atomically dispersed palladium catalyst on layered potassium titanate (KTO), which has enhanced interaction between the TiO_6_ layer and the palladium atoms, is presented. Due to the presence of K^+^ ions in the interlayers of KTO, the TiO_6_ octahedron layers have negative charge, which increases the interaction between Pd atoms and the substrate, thus preventing their agglomeration. In addition, the provision of charge of K^+^ ion makes the molecular oxygen in the system easier to be activated and promotes catalytic oxidation activity.

## Graphical abstract

Due to the presence of K^+^ ions in the interlayers of potassium titanate, the TiO_6_ octahedron layers have negative charge, which is conducive to the atomic dispersion of Pd and the formation of oxygen vacancies. The atomically dispersed palladium catalyst shows enhanced catalytic performance and catalytic stability for room‐temperature catalytic oxidation.

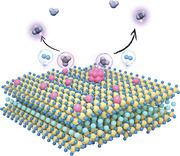

## 1 Introduction

Single‐atom catalysts have high atom utilization and unsaturated coordination environment, thus exhibiting superior activity and selectivity in some important reactions.^[^ ^1^, ^2^, ^3^ ^]^ Obtaining single‐atom catalysts with high loading, high stability, and unique catalytic properties through specific design has always been the core issue.^[^ ^4^ ^]^ Various supports have been used for single‐atom catalysts, such as metal oxides,^[^ ^5^, ^6^, ^7^ ^]^ graphene derivatives,^[^ ^8^, ^9^, ^10^ ^]^ covalent organic framework materials (COFs),^[^ ^11^, ^12^, ^13^ ^]^ and metal–organic framework materials (MOFs).^[^ ^14^, ^15^, ^16^ ^]^ In these systems, unique interaction environments can be ingeniously designed to anchor single atoms on supports, enabling the preparation of single‐atom catalysts. Therein, metal oxides are considered to be excellent supports for the preparation of stable single‐atoms catalysts due to their high mechanical and thermal stability.^[^ ^17^ ^]^ Due to the high surface energy, these atomic metals possess low stability and are prone to agglomeration.^[^ ^18^, ^19^ ^]^ Improving the dispersibility of the loaded metal and increasing the stability of single atoms are key issues for single‐atom catalysts.

Increasing the interaction between the support and the catalyst atoms can improve the dispersion and stability of single‐atom catalysts with metal oxide supports. Specifically, when the interaction between the support and the catalytic atoms is stronger than that of between the catalyst atoms, the agglomeration of the catalyst atoms can be effectively prevented.^[^ ^20^ ^]^ For defect free supports, thermally stable single‐atom catalysts can be prepared when the intrinsic interactions between the supports and the catalytic atoms are strong enough. For example, Pt single atoms can be stabilized on Fe_2_O_3_ supports due to strong interactions.^[^ ^7^ ^]^ In addition, the oxygen vacancies of the metal oxide support can provide uniform action sites for anchoring single atoms, which exhibit unique advantages in the preparation of single‐atom catalysts due to the provision of unsaturated coordination sites.^[^ ^17^ ^]^ Therefore, in order to prepare efficient and stable single‐atom catalysts based on supports, it is necessary to further enhance the intrinsic interactions and increase the number of oxygen vacancies.

In this study, we report the preparation of single atomic Pd on potassium titanate (KTO) nanowires, in which TiO_6_ octahedrons are joined together to form 2D planes stacking layer by layer, between which the K^+^ ions are located in the crystal. Due to the electron donor effect of the interlayer K^+^ ions, such structure can change the charge characteristics of the supports, thereby enhancing the interaction of the Pd atoms and the supports. Therefore, the dispersity and stability of single atom catalysts are improved. In addition, due to the designed charge characteristics of the support, the density of oxygen vacancies also increases. To evaluate the catalytic activity of the prepared Pd/KTO catalysts, the formaldehyde (HCHO) oxidation reaction is chosen as a model reaction, which shows enhanced catalytic activity and excellent stability.

## 2 Results and Discussion

As shown in Figure S1 of the Supporting Information, the synthesized KTO via the direct hydrothermal method exhibits a typical morphology of 1D nanowires with large specific surface area.^[^ ^21^, ^22^, ^23^, ^24^ ^]^ Specially, KTO possesses mixture phases, including layer and tunnel structure (Figure S2, Supporting Information).^[^ ^24^, ^25^, ^26^ ^]^ And the structure of layer potassium titanate (K_2_Ti_4_O_9_) and tunnel potassium titanate (K_2_Ti_6_O_13_) are shown in Figure S3 of the Supporting Information.^[^ ^24^, ^25^, ^26^ ^]^ During the process of deposition–precipitation, K^+^ ions of the layer structure are replaced by H^+^ ions. The escapement of K^+^ from the interlayer leads to the transformation of the layer configuration. Adjacent TiO_6_ layers are separated from each other, and thus the layer‐structure KTO nanowires exfoliate into few‐layered nanosheets, further increasing the specific surface area of the support and providing more deposition sites for the loading metal atoms. The KTO nanosheets with fewer layer is confirmed by the high‐resolution transmission electron microscope (HR‐TEM) image (Figure S4, Supporting Information). Further energy dispersive X‐ray spectroscopy (EDS) during scanning transmission electron microscopy (STEM) characterization shows that the atomic ratio of Ti and K is closer to two, indicating that only a small amount of K^+^ ions escape, and the vast majority of K^+^ ions are still intrinsically located between the support layer (Table S1, Supporting Information). Theoretically, H^+^ can also give negative charge to TiO_6_ octahedron layers. However, during the thermal treatment at 400 °C, the H^+^ in the support escapes as water, leaving behind TiO_2_ (B) (Figure S5, Supporting Information). Therefore, such a negative charge state of the TiO_2_ support surface is not formed. To ensure Pd/KTO catalysts without K^+^ ions on the support surface, repeated cleaning measures by ultrapure water were taken.

**Figure 1:**
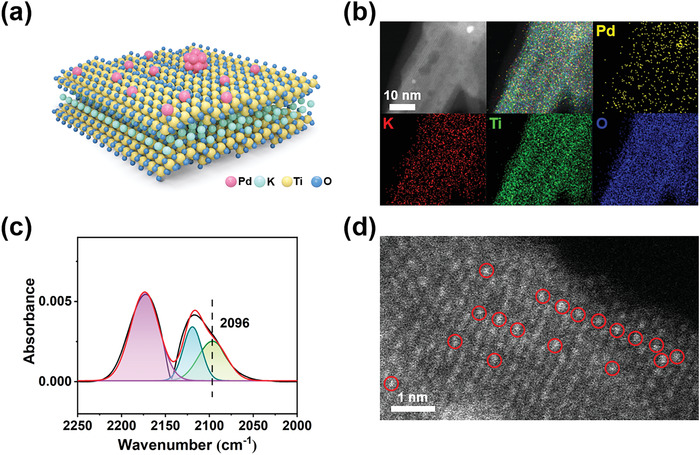
Structural characterizations of the single‐atom Pd_0.1_/KTO catalysts. a) Schematic diagram of single‐atom Pd/KTO catalysts. b) STEM‐EDS elemental mapping of the samples. c) DRIFTS of CO adsorption on the samples. d) HAADF‐STEM image of the samples.

**Figure 2:**
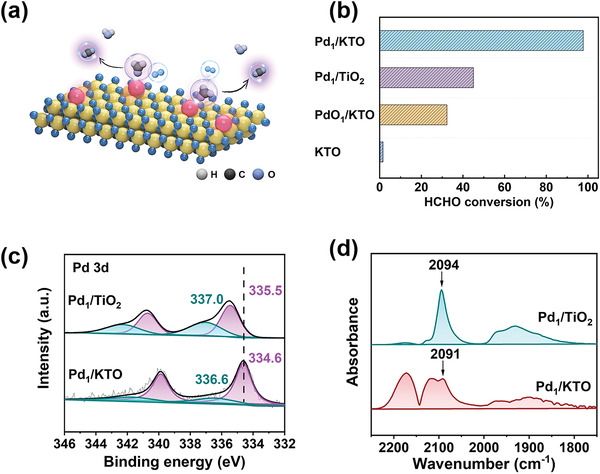
Catalytic performances and characterizations of the catalysts. a) Catalytic mechanism of HCHO oxidation on Pd/KTO. b) Catalytic performances of HCHO oxidation over Pd_1_/KTO, Pd_1_/TiO_2_, PdO_1_/KTO, and KTO. c) XPS spectra of Pd 3d for Pd_1_/TiO_2_ and Pd_1_/KTO. d) DRIFTS of CO adsorption on Pd_1_/TiO_2_ and Pd_1_/KTO.

Generally, high temperature induces the sintering of loading metals, therefore resulting in a negative impact on their catalytic performance. However, it has been reported that the catalytic activity of the obtained Pd/TiO_2_ catalyst calcined at relatively low temperature in H_2_ atmosphere is greatly improved. These enhancements are supposed to be derived from the increase of metal–support interaction, the decrease of the surface Pd particle size and more oxygen vacancies. The Pd_1_/KTO samples with Pd in reduced state can be obtained by calcining in H_2_ atmosphere at 400 °C for 2 h. The structure diagram of Pd/KTO multilayer catalysts is shown in **Figure** **1**a, which can be directly observed in high‐resolution high‐angle annular dark‐field (HAADF)‐STEM (Figure 1b). Most Pd atoms exist in single atom, along with Pd clusters in minority. To further verify that most of the Pd atoms are atomically dispersed in Pd_0.1_/KTO, we performed diffuse reflectance infrared Fourier transform spectroscopy (DRIFTS) of CO adsorption on single‐atom Pd_0.1_/KTO catalysts (Figure 1c). There is a weaker shoulder peak located at 2096 cm^−1^ that can be assigned to the adsorption of CO on single‐atom Pd with a top configuration. And no peak within 2091 cm^−1^ derived from the linear adsorbed CO at Pd atoms is observed, confirming that the Pd atoms are single atomically dispersed on KTO (**Figure** **2**d; Figure S6, Supporting Information).^[^ ^5^, ^27^ ^]^ HAADF‐STEM images of single‐atom Pd/KTO catalysts further exhibit the direct observation of the atomic dispersion of Pd (Figure 1d; Figure S7, Supporting Information).

**Figure 3:**
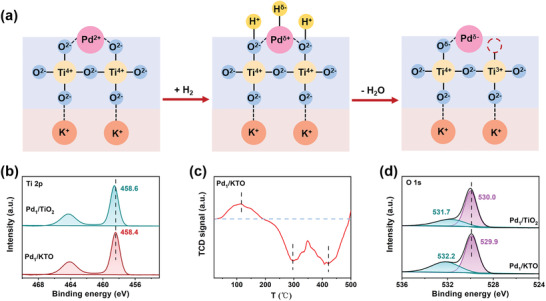
Mechanism and characterization of atomic dispersion of Pd on KTO support. a) Schematic diagram of preparing single‐atom Pd/KTO. b) XPS spectra of Ti 2p in Pd_1_/TiO_2_ and Pd_1_/KTO. c) H_2_‐TPR of fresh Pd_1_/KTO. d) XPS spectra of O 1s in Pd_1_/TiO_2_ and Pd_1_/KTO.

The Coulomb force of K^+^ ions inside the support is considered to be the decisive factor for perpetrating single‐atom Pd/KTO catalysts. Specifically, due to the presence of K^+^ ions in the interlayers of KTO, TiO_6_ octahedron layers have negative charge.^[^ ^28^ ^]^ The surface negatively charge state of KTO has also been confirmed by its isoelectric point.^[^ ^25^, ^29^, ^30^ ^]^ Such negative charge is conducive to the dispersion of Pd precursor and prevents aggregation of Pd precursors after adsorption on the surface of the support. Although Pd^2+^ cation exhibits a stronger coordination ability with Cl^−^ ions.^[^ ^31^ ^]^ However, during the process of preparing catalysts, the Cl^−^ ions of palladium chloride complex will be gradually replaced (Figures S8 and S9, Supporting Information). After the introduction of precipitant, the positively charged Pd ions interact with the oxygen atoms of the support (**Figure** **3**a). Calcinating the obtained Pd/KTO catalyst in H_2_ atmosphere at 400 °C, Pd atoms are still in single atomic dispersion state. In particular, during the process of H_2_ treatment, Pd(II) species are first reduced to Pd(0), and then PdH*_x_* combined with Pd^*δ*+^ and H^*δ*−^ is formed.^[^ ^32^, ^33^ ^]^ The electronic states of Pd atoms annealed in hydrogen atmosphere can be proved by temperature‐programmed reduction of H_2_ (H_2_‐TPR) technique (Figure 3c). With temperature gradually increased from 25 to 500 °C, the consumption of H_2_ was recorded and showed obvious variations. A broad peak between 80 and 120 °C can be observed, refer to the reduction process of Pd(II). In addition, two negative H_2_ consumption peaks characterize the decomposition of the PdH*_x_* phase, suggesting the formation of PdH*_x_* phase during H_2_ treatment. The last H_2_ consumption peak occurs at ≈410 °C, indicating the formation of PdH*_x_* with positively charged Pd. Since the prepared Pd/KTO catalyst is calcined in H_2_ atmosphere at 400 °C, the positively charged Pd is formed. Therefore, due to opposite charge characteristics of Pd species and support surface, there is a strong interaction between the metal and the support. Such interaction prevents the agglomeration of Pd species and stabilizes the dispersed Pd atoms. The smaller average size of Pd clusters in Pd_1_/KTO further confirms the positive role of KTO (Figure S10, Supporting Information).

To further determine the electronic structure of the catalyst, the X‐ray photoelectron spectroscopy (XPS) analysis characterization was carried out. Compared with the Ti 2p binding energy of Pd_1_/TiO_2_ (458.6 eV), the Ti 2p binding energy of Pd_1_/KTO is negatively shifted to 458.4 eV. The lower Ti 2p binding energy of Pd_1_/KTO indicates that KTO has negative charge characteristics compared with TiO_2_ (Figure 3b).^[^ ^34^ ^]^ Due to the negatively charged support, Pd species in reduced states is easier to exist on the support. These results can also be confirmed by the ratio of Pd(II)/Pd(0) species obtained from the XPS spectra of Pd 3d (Figure 2c). The obtained Pd/KTO catalyst not only has stably dispersed single Pd atoms, but also is rich in oxygen vacancies after H_2_ treatment. The properties of oxygen vacancies can also be verified by XPS characterization. Specifically, as shown in Figure 3c, compared with Pd_1_/TiO_2_ catalysts, the amount of adsorbed oxygen on the Pd_1_/KTO is more, indicating the higher density of oxygen vacancies in the KTO support.^[^ ^35^ ^]^ In generally, for oxide supports, reductive treatment is helpful in the formation of oxygen vacancies.^[^ ^17^ ^]^ The negative charge on the support surface is in favor of the reduction of KTO support, resulting in more oxygen vacancies. This defect of support not only helps to disperse single atomic metal catalyst, but also captures oxygen species to improve the performance of the catalysts.

Formaldehyde (HCHO) is a noxious pollute of the indoor environment, emitting commonly from widely used building and decorative materials, which would induce serious health problems even at very low concentration (lower than ppm).^[^ ^1^ ^]^ Catalytic oxidation of HCHO to nontoxic CO_2_ and H_2_O is considered to be one of the most promising method to lower indoor HCHO concentration. Supported Pd is an efficient catalyst for catalytic oxidation of HCHO at room temperature. Therefore, HCHO oxidation reaction is selected as the key reaction to evaluate the catalytic activity of newly prepared Pd/KTO catalysts (Figure 2a). In this process, HCHO is oxidized and decomposed into CO_2_ and H_2_O. Compared with Pd_1_/TiO_2_ catalysts, Pd_1_/KTO catalysts exhibit an extremely high activity and stability (Figure 2b). A HCHO conversion larger than 98% is achieved over Pd_1_/KTO, while a lower conversion of 45% is obtained with Pd_1_/TiO_2_ catalyst.

**Figure 4:**
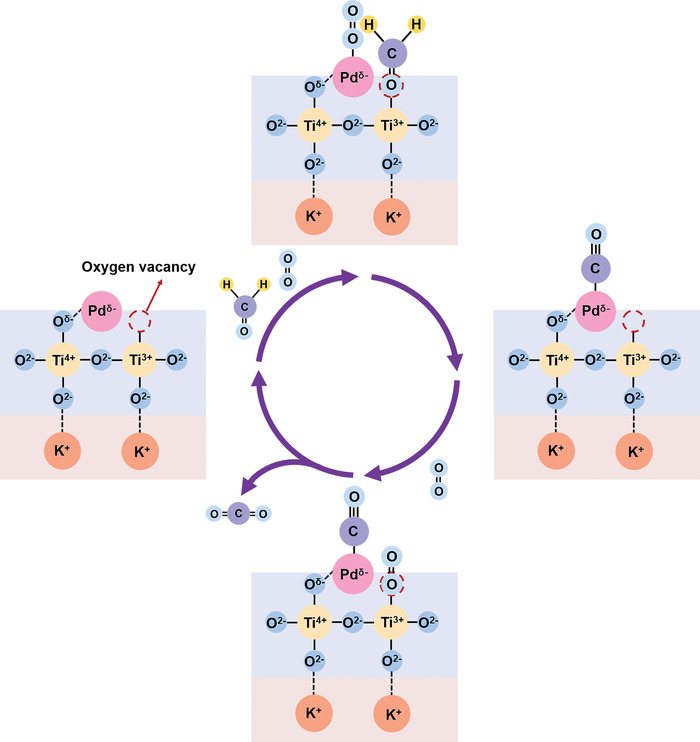
Catalytic mechanism of HCHO oxidation on Pd/KTO.

Such high catalytic activity of Pd_1_/KTO can be attributed to the presence of KTO support. As discussed above, the existence of K^+^ ions makes the KTO surface more negatively charged. Due to the strong interaction, the charge further transfers from KTO surface to the Pd, resulting in an increase in the electron density of Pd. The impact of support on the electronic structure of Pd species can be proved by XPS analysis of Pd 3d on the Pd_1_/KTO and Pd_1_/TiO_2_. The Pd(0) binding energies of Pd_1_/KTO shift to lower values, suggesting that the Pd species are more negatively charged on KTO than those on TiO_2_ (Figure 2c). The presence of negatively charged Pd species can also be confirmed by the DRIFTS of CO adsorption (CO‐DRIFTS), because when CO is chemically adsorbed on the metal, the d–*π* feedback between the metal and CO can suggest the electronic structure of the loaded metal.^[^ ^36^, ^37^ ^]^ The wavenumber of linear adsorbed CO bonds at Pd atoms of Pd_1_/KTO (2091 cm^−1^) is lower than that of Pd_1_/TiO_2_ (2094 cm^−1^), indicating that more electrons are fed back to the CO molecule (Figure 2d). Such high feedback electron is consistent with supposing that Pd species are more negatively charged in Pd_1_/KTO (Figure 1c). In addition, CO oxidation is considered to be the key process of HCHO oxidation. More electrons feedback from metal to CO implies higher reactivity of CO oxidation, which also indicates that the catalysts have high catalytic activity for HCHO oxidation. PdO/KTO catalyst with preadsorbed oxygen exhibits much poorer conversion of 37% than that of the pristine Pd_1_/KTO (98%). The great difference in activity between Pd_1_/KTO and PdO/KTO indicates the decisive role of reduced Pd species for the catalytic performance. When the PdO_1_/KTO sample is retreated in H_2_, the sample gives improved HCHO conversion (78%), which is also more active than Pd_1_/TiO_2_ (Figure S11, Supporting Information). The result further indicates the high charge density of Pd leads to the high catalytic activity for HCHO oxidation (**Figure** **4**).

As mentioned above, the high activity comes from the highly dispersed Pd catalyst and the rich oxygen vacancies of KTO (Figure 4). During HCHO oxidation reaction, HCHO is introduced into the system by flowing N_2_ gas in advance and adsorbs on the oxygen vacancies of KTO. Therefore, due to the lack of unoccupied oxygen vacancy sites, later introduced oxygen adsorbs on Pd species, which can be proved by the content of PdO by XPS characterization. As discussed above, the presence of intrinsic K^+^ ions in KTO prevents the agglomeration of Pd and enhances the negative charge of Pd atoms/clusters. Thus, compared with Pd_1_/TiO_2_, Pd atoms and smaller Pd clusters exist in obtained Pd_1_/KTO catalyst. Such smaller Pd species have more abundant active sites, which are favorable for the dissociative adsorption of oxygen on the catalyst surface. In addition, negatively charged Pd is conducive to the activation of oxygen, which enhances the feedback between the Pd and the π* orbital of O_2_, further promoting the absorption of O_2_.^[^ ^34^ ^]^ And higher electrons fed back improve the activation of O_2_. And then CO intermediate generated by the decomposition is readsorbed on Pd, and molecular oxygen adsorbs on the released oxygen vacancy sites. Therefore, the rich oxygen vacancies and negatively charged surface of KTO further promote the oxidation of HCHO.

## 3 Conclusion

In summary, based on the interaction between the KTO support and the loaded Pd, a convenient strategy for constructing atomically dispersed palladium catalyst is proposed. The negativity charged TiO_6_ layers alternated with interlayer K^+^ ions are the key to this strategy. On the one hand, such negatively charged TiO_6_ layer is beneficial to the dispersion of Pd during deposition–precipitation process and the stable existence of Pd atoms during the high temperature hydrogen treatment process. On the other hand, such negatively charged TiO_6_ layer is conducive to the formation of oxygen vacancies. Therefore, single‐atom dispersed Pd is achieved on the obtained Pd_1_/KTO catalyst. Both negatively charged Pd and abundant oxygen vacancies contribute to the enhanced catalytic performance of the catalyst to room‐temperature HCHO oxidation, which provides a new route to construct efficient noble‐metal single‐atom catalysts.

## 4 Experimental Section

### Materials

Commercial P25 (Evonik, 50 m^2^ g^−1^, 80% anatase, 20% rutile) powder was purchased from Degussa AG. PdCl_2_ (AR), CO(NH_2_)_2_ (AR), NaOH (AR), and KOH (AR) were purchased from Sinopharm Chemical Reagent Co., Ltd.

Synthesis of KTO. KTO was synthesized by a typical hydrothermal process.^[^ ^24^ ^]^ Specifically, P25 of 2.5 g was added into 500 mL KOH aqueous solution (10 m) under stirring. The mixture was placed in Teflon autoclave and then kept at 200 °C for 6 days. After the reaction, the obtained powder was collected through suction filtration, and then washed with ultrapure water several times until the filtrate became neutral. After drying, KTO was acquired.

Synthesis of Pd_1_/KTO nanostructure. Pd_1_/KTO nanomaterials were prepared by deposition–precipitation method with PdCl_2_ as precursor (10 g L^−1^). Under stirring, KTO of 0.1 g was evenly dispersed in 50 mL aqueous solution containing 166 µL PdCl_2_. Then, 100 mL 0.25 m urea was dropped into aqueous solution as precipitant. Then, the suspension was stored in a constant temperature of 80 °C water under vigorous magnetic stirring in dark. After 4 h, the resulting precipitate was filtered and washed with ultrapure water several times. The filter cake was dried in air at 80 °C for 12 h. The prepared catalysts were then treated at 400 °C for additional 2 h with a 5 °C min^−1^ heating rate, and the sample marked as Pd_1_/KTO.

Synthesis of Pd_0.1_/KTO nanostructure. Pd_0.1_/KTO nanomaterials were prepared by deposition–precipitation method with PdCl_2_ as precursor (10 g L^−1^). Under stirring, KTO of 0.1 g was evenly dispersed in 50 mL aqueous solution containing 16.6 µL PdCl_2_. Then, 100 mL 0.25 m urea was dropped into aqueous solution as precipitant. Then, the suspension was stored in a constant temperature of 80 °C water under vigorous magnetic stirring in dark. After 4 h, the resulting precipitate was filtered and washed with ultrapure water several times. The filter cake was dried in air at 80 °C for 12 h. The prepared catalysts were then treated at 400 °C for additional 2 h with a 5 °C min^−1^ heating rate, and the sample marked as Pd_0.1_/KTO.

Synthesis of H_2_Ti_3_O_7_ (HTO). Specifically, P25 of 2.5 g was added into concentrated 500 mL NaOH aqueous solution (10 m) under stirring. The mixture was placed in Teflon autoclave and then kept at 200 °C for 3 days. After the reaction, the obtained powder was collected through suction filtration, and then washed with ultrapure water several times till neutral. After drying, Na_2_Ti_3_O_7_ was acquired. The as‐prepared Na_2_Ti_3_O_7_ was acidified in 0.1 m HCl for 24 h, then thoroughly washed with deionized water and dried to obtain HTO.

Synthesis of Pd_1_/TiO_2_ nanostructure. The Pd_1_/TiO_2_ nanomaterials were prepared through the same process as the preparation of Pd_1_/KTO, in the process of using HTO as support.

### Characterizations

Powder X‐ray diffraction analysis of the samples were carried out with a PANalytical X'pert3 powder diffractometer (40 kV, 40 mA) using Cu K*α* radiation. The patterns were recorded in the range of 10°–80° with a step size of 0.013°. Scanning electron microscope and energy dispersion spectrum (EDS) were obtained on a Zeiss Gemini300 scanning electron microscope. TEM and HR‐TEM images were obtained with a JOEL JEM 2100 microscope. HAADF‐STEM images were acquired on a Titan Cubed Themis G2 300. XPS measurements were analyzed using a Thermo ESCALAB 250 X‐ray photoelectron spectrometer, and the binding energies were determined utilizing C 1s spectrum as reference at 284.8 eV. DRIFTS measurements were carried out on a BRUKER VERTEX‐70 FTIR. H_2_‐TPR was conducted on a Builder PCSA‐1000 instrument.

### Catalytic Activity Test

Before catalytic test and characterization, the as‐synthesized samples Pd/KTO and Pd/TiO_2_ were treated under H_2_ flow with a 5 °C min^−1^ heating rate at 400 °C for 2 h, and marked as Pd_0.1_/KTO, Pd_1_/KTO and Pd_1_/TiO_2_, respectively. Before the characterization, the Pd/TiO_2_ catalyst was first reduced with H_2_ at 300 or 450 °C for 1 h, followed by purging with He for 30 min, and then the system was cooled down to 25 °C. Typically, for H_2_‐TPR, the fresh samples were pretreated by He for 30 min, and then purged with He for 30 min at 25 °C. Then, the system was cooled down to −50 °C followed by reduction with 10% H_2_/Ar from −50 to 100 °C at a rate of 10 °C min^−1^. The samples pretreated in oxygen are denoted as PdO/KTO. The samples of 50 mg were carried out in a quartz tubular (i.d. = 6 mm) fix bed reactor under normal pressure at room temperature. Gaseous HCHO was produced by flowing N_2_ via pyrolysis of paraformaldehyde held at 28 °C under water bath. The feed gas containing 200 ppm HCHO and simulated air (O_2_/N_2_ = 21:79). The total flow rate was 50 mL min^−1^. The flow rate of the gas was regulated by a mass flow controller. The HCHO concentration in feed gas and exit gas was determined by the phenol reagent colorimetric method (GBT18204.2‐2014, China).

The conversion of HCHO can be expressed as

(1) $$\mathrm{HCHO}\;\mathrm{conversion}\left(\%\right)=\frac{{\left[\mathrm{HCHO}\right]}_{\mathrm{in}}-{\left[\mathrm{HCHO}\right]}_{\mathrm{out}}}{{\left[\mathrm{HCHO}\right]}_{\mathrm{in}}}\times 100\%$$

where [HCHO]_in_ and [HCHO]_out_ is the HCHO concentration in feed gas and product gas stream, respectively.

## Conflict of Interest

The authors declare no conflict of interest.

## Supporting information

Supporting Information

## Data Availability

The data that support the findings of this study are available in the Supporting Information of this article.
