# Supplementary material for: Potassium Titanate Supported Atomically Dispersed Palladium for Catalytic Oxidation
Source: Adv Sci (Weinh). 2022 Oct 26;10(2):2204674. doi: 10.1002/advs.202204674 (PMC9839854; doi:10.1002/advs.202204674)
Supplement: Supplementary file 1 — Supporting Information [file ADVS-10-2204674-s001.pdf]

ADVANCED SCIENCE
Open Access

# Supporting Information

for *Adv. Sci.*, DOI 10.1002/advs.202204674

Potassium Titanate Supported Atomically Dispersed Palladium for Catalytic Oxidation

*Li Zhou, Shuren He, Xiaohong Xu*, Guangwu Li* and Chuancheng Jia**

Supporting Information for

# Potassium Titanate Supported Atomically Dispersed Palladium for Catalytic Oxidation

*Li Zhou, Shuren He, Xiaohong Xu,* Guangwu Li,* and Chuancheng Jia**

L. Zhou, Prof. G. Li*, Prof. C. Jia*

Center of Single-Molecule Sciences, Institute of Modern Optics, Tianjin Key Laboratory of Micro-scale Optical Information Science and Technology, Frontiers Science Center for New Organic Matter, College of Electronic Information and Optical Engineering, Nankai University, 38 Tongyan Road, Jinnan District, Tianjin 300350, P. R. China.

E-mail: jiacc@nankai.edu.cn; ligw@nankai.edu.cn

S. He, Prof. X. Xu*

School of Chemistry and Chemical Engineering, Shandong University, 27 Shanda Nan Road, Licheng District, Jinan, Shandong 250100, P.R. China

E-mail: xhxu@sdu.edu.cn

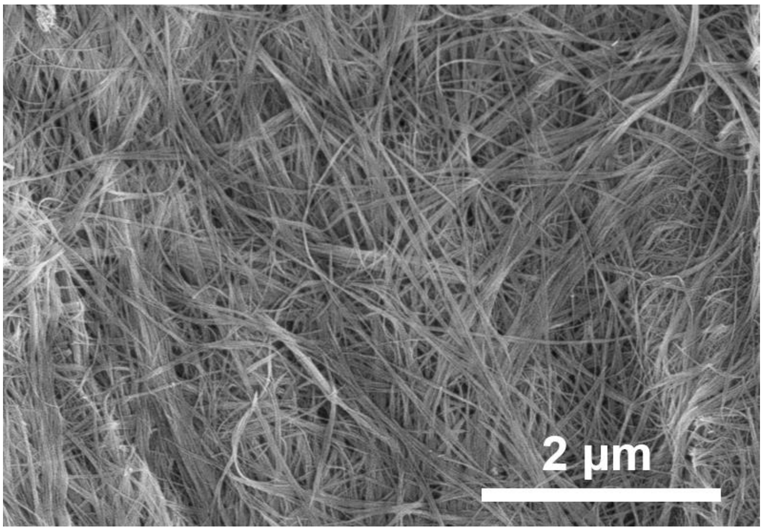


**Figure S1.** Representative scanning electron microscope (SEM) image of KTO nanowires. KTO nanowires prepared by hydrothermal method show typical one-dimensional morphology, with tens of micrometers in the axial direction.

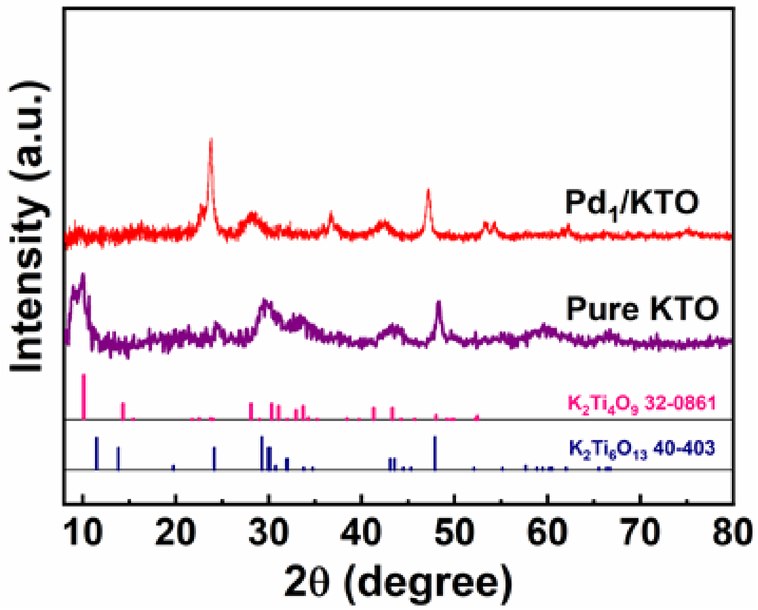


**Figure S2.** The XRD patterns of $Pd_1$/KTO and pure KTO. The XRD of pure KTO shows that it has mixture phases, including layer structure and tunnel structure.[1-3] For $Pd_1$/KTO, there is phase transformation due to the thermal treatment in $H_2$ at 400°C.

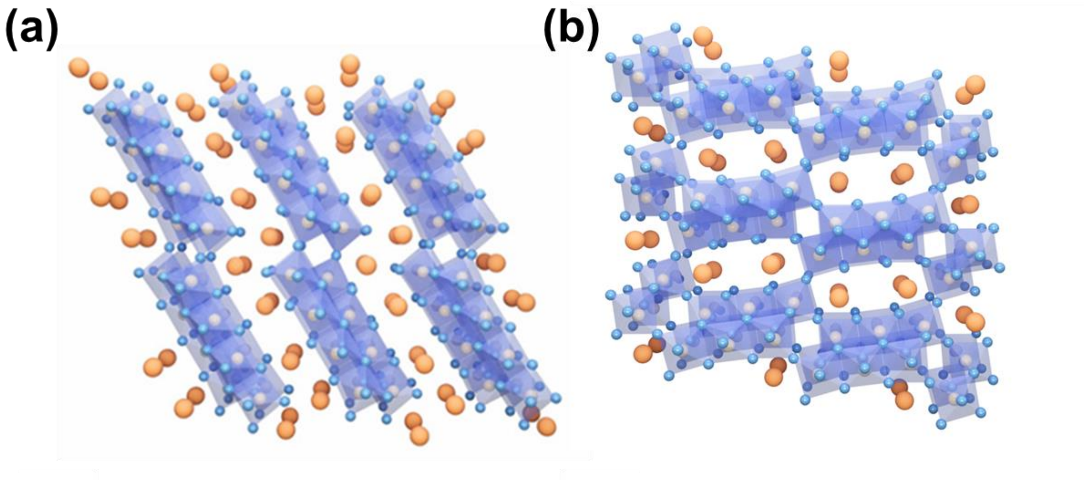


**Figure S3.** Structure diagram of (a) layer potassium titanate ($K_2Ti_4O_9$) and (b) tunnel potassium titanate ($K_2Ti_6O_{13}$). The light brown, blue and orange balls represent K, O and Ti atoms, respectively.

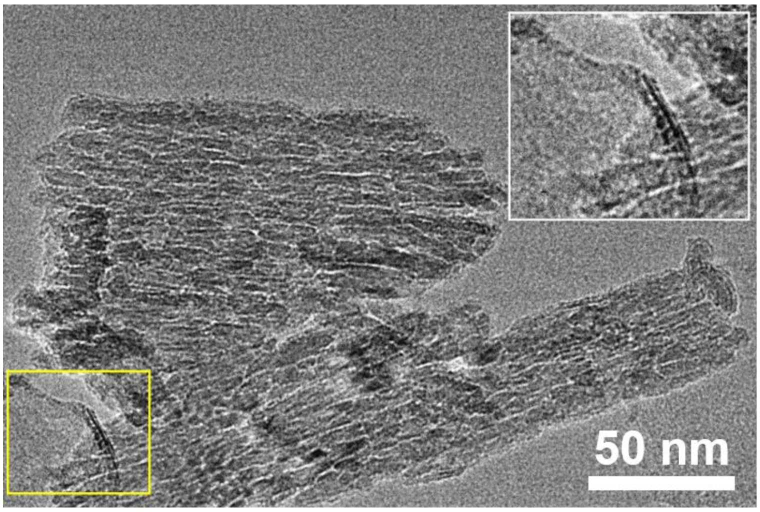


**Figure S4.** Representative transmission electron microscopy (TEM) image of KTO nanowires. The layered KTO nanowires are exfoliated into flakes, further increasing the specific surface area of the support and providing more deposition sites for the loaded metal.

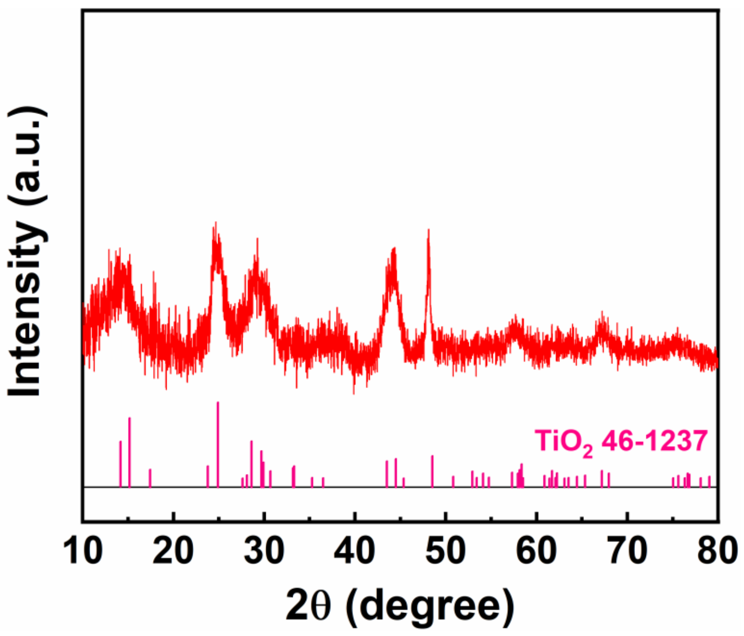


**Figure S5.** The XRD patterns of $H_2Ti_3O_7$ after calcination at 400°C.

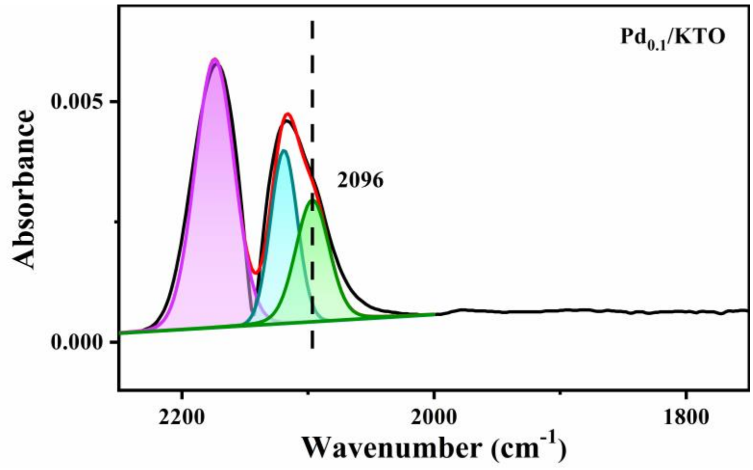


**Figure S6.** Diffuse reflectance infrared Fourier transform spectroscopy (DRIFTS) of CO adsorption on single-atom $Pd_{0.1}$/KTO catalysts with larger range. The peak located at 2096 $cm^{-1}$ indicates the adsorption of CO on single-atom Pd with a top configuration. There is no peak in the range of 2000~1800 $cm^{-1}$, which confirms that Pd atoms are highly dispersed.

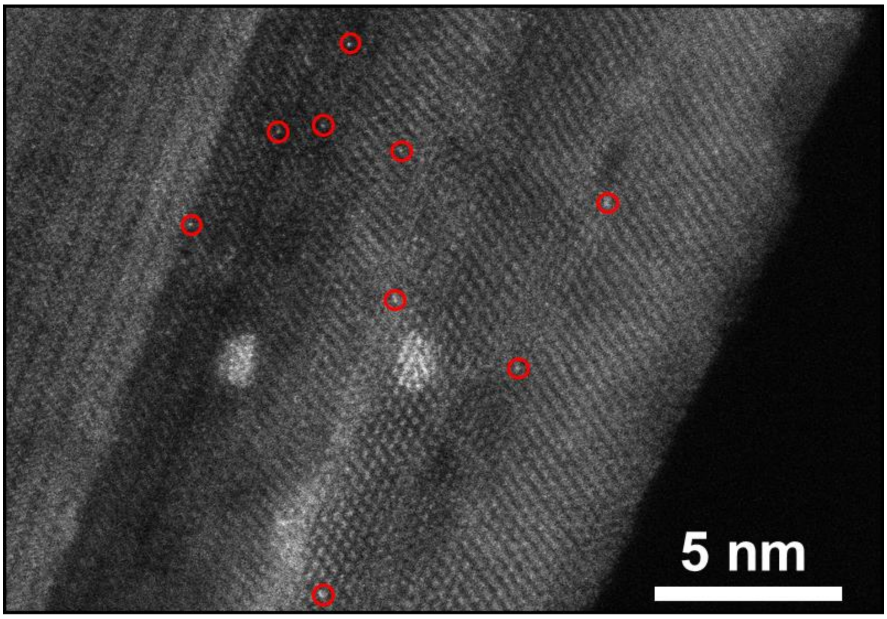


**Figure S7.** High-resolution high-angle annular dark-field (HAADF) STEM image of single-atom $Pd_1$/KTO catalysts.

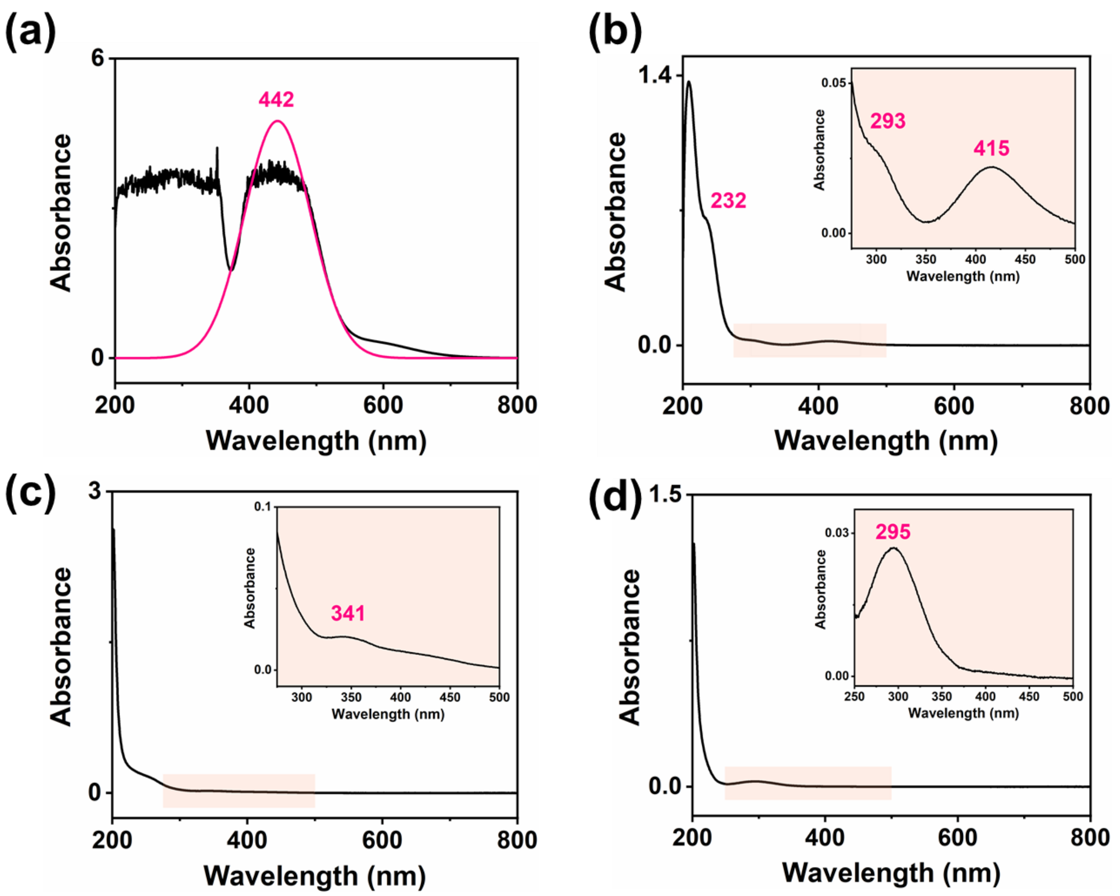


**Figure S8.** The UV/Vis absorption spectra of aqueous solution of (a) 10 g/L $PdCl_2$ precursor solution, (b) M1, (c) M2 and (d) M2 after heating at 80°C for 2 h.

During preparing catalysts, the $PdCl_2$ precursor solution was first uniformly dispersed in the solution, and then deposited on the surface of the support under the effect of the precipitant. Therefore, four samples are chosen to analyze by UV/Vis, including $PdCl_2$ precursor solution (10 g/L, solid palladium chloride dissolved in 0.1 M hydrochloric acid solution), dilution solution of precursor (166 μL $PdCl_2$ precursor solution is added to 50 mL ultrapure water, and then evenly dispersed, the mixture is recorded as M1), the mixture of M1 and urea solution (add 100 ml 0.25 M urea solution to the M1 solution, the mixture is recorded as M2), and the solution that M2 after heating at 80°C for 2 h.

According to the analysis from Figure R2, the $PdCl_2$ precursor solution has been hydrolyzed (Figure S8a). Although the concentration of $PdCl_2$ precursor solution above the detection limit, a peak at 442 nm can be fitted by Gaussian analysis, indicating the mixture of $PdCl_4^{2-}$ and $PdCl_3(H2O)^-$.[4] After dilution with ultrapure water, the hydrolysis of the palladium chloride complex is further promoted due to the decrease of $Cl^-$ and $H^+$

concentration. It is shown in Figure S8b, the peak occurs at 415 nm, which can be attributed to the combined action of $PdCl_2(H_2O)_2$ and $PdCl(H_2O)_3^+$.[4] Since the $H^+$ of solution promotes the hydrolysis of urea, the $Cl^-$ are further substituted with the adding of urea solution. Therefore, there is $[Pd(NH_3)_2(H_2O)_2]^{2+}$ of M2 (Figure S8c).[5] After heating at 80°C for 2 h, the hydrolysis of urea is enhanced, the $Cl^-$ in the Pd complex are completely replaced. As shown in Figure R2d, the peak at 295 nm can be attributed to $[Pd(NH_3)_4]^{2+}$.[6]

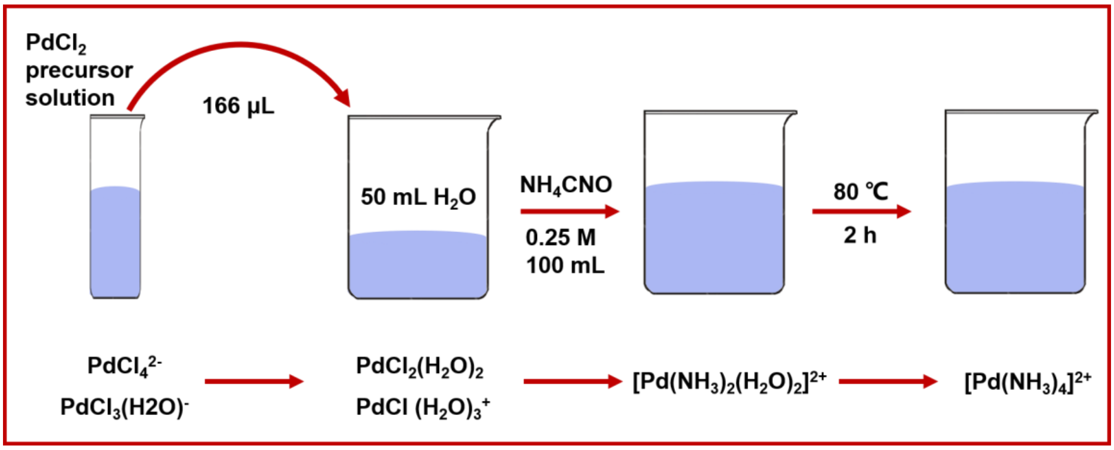


**Figure S9.** Schematic of structural changes of Pd complexes in the solution. The structure changes of Pd complexes are summarized in the schematic.

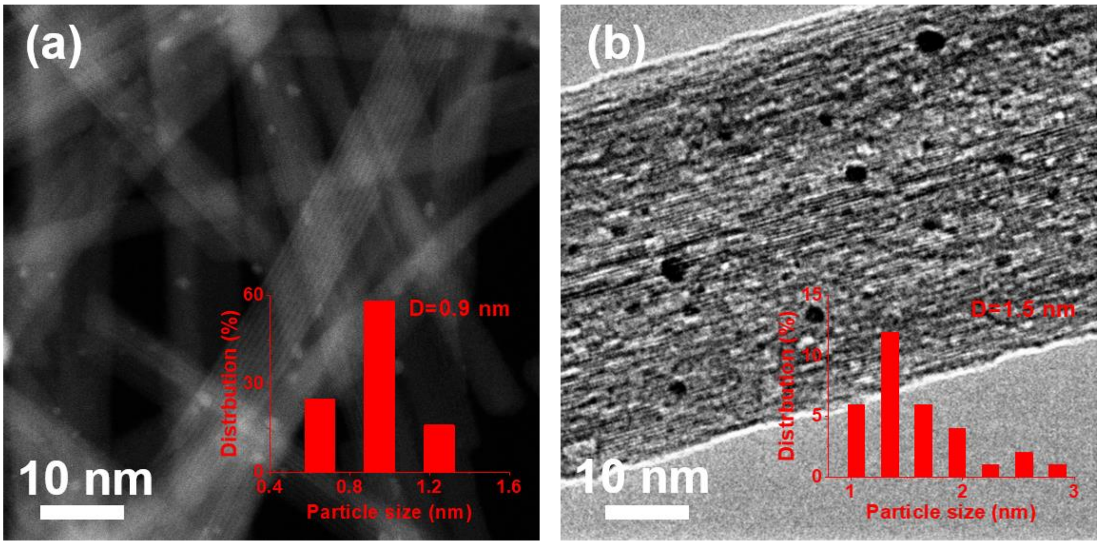


**Figure S10.** Typical TEM images and the corresponding cluster size distributions of as-prepared $Pd_1$/KTO and $Pd_1/TiO_2$.The Pd clusters of $Pd_1$/KTO show a smaller average size of 0.9 nm, which is less than 1.5 nm of $Pd_1/TiO_2$. The difference in the average size of Pd clusters proves the promoting effect of KTO on the dispersibility of the loaded Pd.

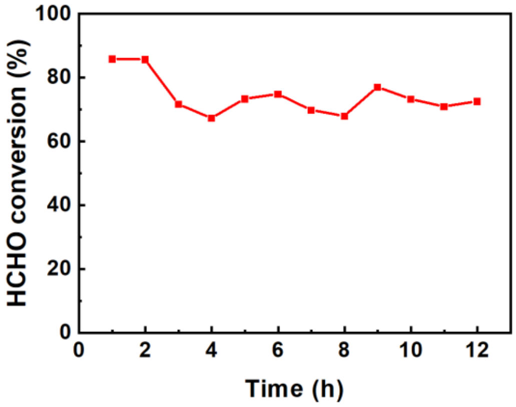


**Figure S11.** The HCHO oxidation over the sample that $PdO_1$/KTO reduction in $H_2$ at 400℃ for 2 h.

**Table S1.** Energy dispersion spectrum (EDS) spectrum of KTO.

| Element | Norm. C (wt.%) | Atom. C (wt.%) |
|---|---|---|
| O | 41.42 | 66.69 |
| Ti | 43.80 | 23.56 |
| K | 14.79 | 9.74 |
| Pd | 0.00 | 0.00 |
| | 100 | 100 |

**References**

[1] A. A. Jeffery, A. Pradeep, M. Rajamathi, *Phys. Chem. Chem. Phys.* **2016**, *18*, 12604.

[2] K. S. Yun, J. Choi, I. Hwang, S. Hong, B. H. Park, *J. Korean Phys. Chem.* **2008**, *52*, 466.

[3] D. Kapusuz, Y. E. Kalay, J. Park, A. Ozturk, *J. Ceram. Process. Res* **2015**, *16*, 291.

[4] L. Espinosa-Alonso, K. P. de Jong, B. M Weckhuysen, *Phys. Chem. Chem. Phys.* **2010**, *12*, 97.

[5] K. Shimizu, R. Maruyama, S. Komai, T. Kodama, Y. Kitayama, *J. Catal.* **2004**, *227*, 202.

[6] B. S. Lalia, N. Yoshimoto, M. Egashira, M. Morita, *J. Power Sources* **2010**, *195*, 7246.
